# Effects of Bubbles During Water Resistance Therapy on the Vibration Characteristics of Vocal Folds During the Phonation of Different Vowels

**DOI:** 10.3390/jcm15020669

**Published:** 2026-01-14

**Authors:** Marie-Anne Kainz, Rebekka Hoppermann, Theresa Pilsl, Marie Köberlein, Jonas Kirsch, Michael Döllinger, Matthias Echternach

**Affiliations:** 1 Division of Phoniatrics and Pediatric Audiology, Department of Otorhinolaryngology, University Hospital, Ludwig-Maximilian-University Munich, Marchioninistrasse 15, 81377 Munich, Germany; marieanne.kainz@med.uni-muenchen.de (M.-A.K.); rebekka.hoppermann@med.uni-muenchen.de (R.H.); theresa.pilsl@med.uni-muenchen.de (T.P.); marie.koeberlein@med.uni-muenchen.de (M.K.); jonas.kirsch@med.uni-muenchen.de (J.K.); 2 Clinic for Anesthesiology and Intensive Care Medicine at Charité, University Medicine Berlin, Charitéplatz 1, 10117 Berlin, Germany; 3 Antonio Salieri Department of Vocal Studies and Vocal Research in Music Education, mdw University of Music and Performing Arts Vienna, Anton-von-Webern-Platz, 1030 Vienna, Austria; 4 Division of Phoniatrics and Pediatric Audiology, Department of Otorhinolaryngology Head & Neck Surgery, University Hospital Erlangen, Friedrich-Alexander-University Erlangen-Nürnberg, Waldstrasse 1, 91054 Erlangen, Germany; michael.doellinger@uk-erlangen.de

**Keywords:** voice, semi occlusion, therapy, water resistance therapy

## Abstract

**Background:** Semi-occluded vocal tract exercises (SOVTE) improve vocal quality and capacity. Water resistance therapy (WRT), a specific form of SOVTE with a tube submerged under water, generates increased and oscillating oral pressure through bubble formation during phonation, thereby influencing transglottal pressure and vocal fold dynamics. While the physiological effects of WRT using tube-based systems have been extensively studied, the influence of vowel-specific vocal tract configurations during WRT remains unclarified. This study examined how different vowel qualities during WRT affect vocal fold oscillation using the DoctorVox^®^ mask, which allows near-natural mouth opening and vowel articulation. **Methods:** Ten vocally healthy, untrained adults (25–50 years) performed a continuous vowel glide (/i/–/a/–/u/-/i/) at constant fundamental frequency and habitual loudness during WRT using the DoctorVox^®^ mask, with the tube submerged 2 cm in water. Simultaneous recordings included transnasal high-speed videoendoscopy (20,000 fps), electroglottography (EGG), acoustic signals and intra-tube oral pressure measurements. Glottal area waveforms (GAW) were derived to calculate the open quotient (OQ_GAW_) and closing quotient (ClQ_GAW_). Analyses were conducted separately for intra-tube pressure maxima, minima and intermediate phases within the bubble cycle during WRT. Statistical analysis used Wilcoxon signed-rank tests with Bonferroni correction. **Results:** In the baseline condition without WRT, significant vowel-related differences were found: /u/ showed a higher open quotient than /i/ and /a/ (*p* < 0.05) and a higher closing quotient than /a/ (*p* < 0.05). During WRT, these vowel-specific differences were no longer statistically significant. A non-significant trend toward reduced OQ_GAW_ during WRT was observed, most notably for /u/, while differences between pressure phases within the bubble cycle were minimal. **Conclusions:** WRT using the DoctorVox^®^ mask reduces vowel-specific differences in vocal fold vibration patterns, suggesting that for voice therapy, vowel quality modifications during WRT have little impact on vocal outcomes.

## 1. Introduction

Over the past decades, there has been a growing understanding of non-surgical voice therapies in patients suffering from dysphonia. In this respect, an increasingly common voice and speech therapy approach known as semi-occluded vocal tract exercises (SOVTE) has been shown to be beneficial for vocal quality and capacity, in which a semi-occlusion at the lips or by adding tube systems influences the transglottal pressure (P_trans_) difference and thus vocal fold oscillations [1].

A specific type of SOVTE is water resistance therapy (WRT) in which the tube does not end in free air but is submerged in water. This therapy has been shown to have positive effects on vocal health in various groups, such as healthy individuals, subjects with functional/maladaptive voice disorders or dysphonia with mass lesions and singers [2,3,4,5,6,7].

Due to the immersion depth of the tube during WRT, the intraoral pressure (P_oral_) increases, changing the P_trans_ gradient during phonation, which is the main source of voice production due to the establishment of vocal fold oscillations and the initiation of transglottic flow [8,9]. It has been shown that subglottal pressure (P_sub_) is upregulated during WRT and that the open phase is broader during the oscillation due to the general increase in pressure [10,11]. It could be hypothesized that the latter leads to a stretching effect on the vocal folds, but also reduces collision pressures during vocal fold closure, thus reducing the risk of voice strain during the exercises. In WRT, the pressure is repeatedly relieved by the formation of bubbles, resulting in intraoral pressure fluctuations (oscillations of P_oral_) that not only influence the vibratory function of the vocal folds, but also presumably have a massage-like effect in the vocal tract [10,12,13]. Numerous studies have examined various factors influencing WRT on the vocal tract and phonation. For example, it has been shown that the shallower the tube was immersed in water, the more stable the phonation remained [14]. The influence of different frequencies of bubbles and changes in the amplitude of P_oral_ oscillations was also examined in more detail. Tyrmi et al. concluded that higher amplitudes of oral oscillations (peak-to-peak P_oral_) may have a positive impact on the massage effect during WRT [15]. Guzman et al. observed that during WRT through pipes with a wider diameter, there was a more regular and lower bubble frequency and a higher bubble amplitude, which led to a higher subjective massage sensation (vibrations during bubbling) [16].

Although there were general positive effects of this approach for long-term training, the short-term effects of WRT were found to be rather low. In this respect, it has been shown that for healthy voices there was a short-term decrease in perturbation values, five minutes after a ten-minute WRT [17]. For patients with vocal fold mass lesions, there was an increase in the glottal area-derived open quotient immediately after a ten-minute intervention [4].

Most of the studies concerning WRT have been performed during phonation in tube systems such as LaxVox^®^ or glass tubes. All these methods limit the mouth opening to a rather unnatural, mostly narrow opening, depending on the diameter of the tube. In contrast, the DoctorVox^®^ mask has been introduced as a mask combined with a tube which allows almost natural mouth openings during WRT [18]. Studies on voice therapy with semi-occluded ventilation masks (SOVM) but without water resistance have shown positive effects on vocal parameters and subjective self-assessment directly after exercising [6,19]. However, there are no studies on vocal parameters during the SOVM exercises.

The DoctorVox^®^ mask combines a mask and WRT and allows for adjusting to the vocal tract shape according to rather natural positions for any vowel. However, the different vocal tract shapes for vowels could have a strong effect on the P_trans_ difference as well as on the vocal fold oscillation patterns during WRT using the DoctorVox^®^ mask.

To date, it has not been clarified how the vocal tract shape for different vowel qualities influences the WRT effect on vocal fold oscillatory dynamics. Further, it remains unclear how the magnitude of the bubble influences such vocal fold oscillations and whether bubble size is vowel dependent. The aim of the presented study was to examine these aspects using high-speed laryngoscopy (HSV), electroglottography and audio signals.

## 2. Materials and Methods

After approval from the local ethics committee (Medical Ethics Committee of the University of Munich 3.9.2020, (No.20/282.)), 10 healthy, vocally untrained subjects (age range 25–50 years; 5 female, 5 male) were included (Table 1). For this prospective study the exclusion criteria included age < 18 years and age > 65 years, excessively narrowed nasal passages, laryngeal or vocal tract pathology and dysphonia. None of the participants reported self-perceived voice problems. Vocal fold mass lesions were excluded via evaluation using videostroboscopy or high-speed video laryngoscopy (HSV), respectively, by an experienced phonatrician and laryngologist. Furthermore, subjective voice problems were excluded using the Voice Handicap Index in German translation (VHI) [20].

**A. Task**

**Figure 1:**
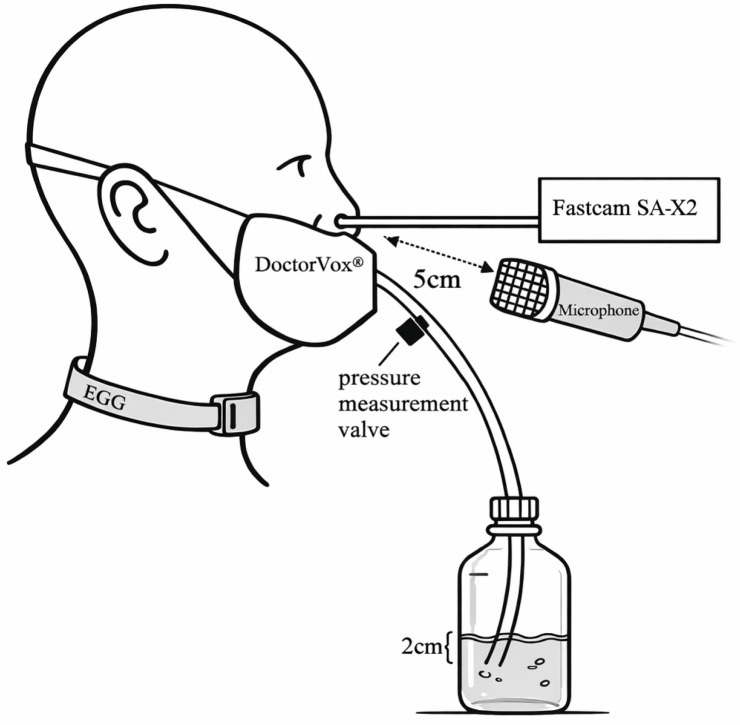
Schematic representation of the experimental setup.

All participants were asked to perform a vowel glide from /i/ to /a/ to /u/ to /i/ on fundamental frequency (ƒ_o_), males ≈ 125 Hz and females ≈ 250 Hz, at habitual loudness during a WRT using the DoctorVox^®^ mask. (DoctorVox, Ankara, Turkey) The immersion depth was fixed at 2 cm below the water surface (Figure 1). Each vowel should be sustained for approximately one second, and the change in vowel quality should be performed as smoothly as possible. In order to avoid habituation effects, there was only one take per subject.

**B. Recordings**

The participants’ phonations were recorded simultaneously using transnasal HSV, electroglottography (EGG) and a microphone, following procedures established in previous studies [4,17]. While HSV provides a two-dimensional endoscopic view of the vocal folds, EGG captures their three-dimensional contact behavior by measuring variations in electrical impedance.

HSV data were acquired transnasally with a Fastcam SA-X2 system (Photron, Tokyo, Japan) coupled to a flexible endoscope (ENF-GP, Olympus, Hamburg, Germany) at 20,000 frames per second and an image resolution of 386 × 320 pixels. Acoustic signals were recorded using a DPA 4061 microphone (DPA Microphones, Kokkedal, Denmark). Sound pressure level calibration (measured over time) was performed using the Sopran software (Svante Granqvist, version 1.0.34) in combination with a sound level meter (Voltcraft, Taipe, Taiwan). EGG signals were captured with an EG2-PCX2 device (Glottal Enterprises, Syracuse, NY, USA). All data was recorded at 20,000 frames per second.

Additionally, pressure inside the tube between the mask and the water reservoir was measured using a PT70 sensor connected to an MS 110 unit (Glottal Enterprises, Syracuse, NY, USA). Calibration was conducted with a PC-1 calibrator (Glottal Enterprises, Syracuse, NY, USA). No anesthetic agents were administered for the transnasal endoscopic procedure.

HSV recordings were post-processed-rotated, Fourier-filtered and cropped. From HSV recordings, the glottal area waveform (GAW) and phonovibrograms [21] were computed using the Glottis Analysis Tools Software (GAT) version 2020 [22].

**C. Bubble Cycle analysis**

**Figure 2:**
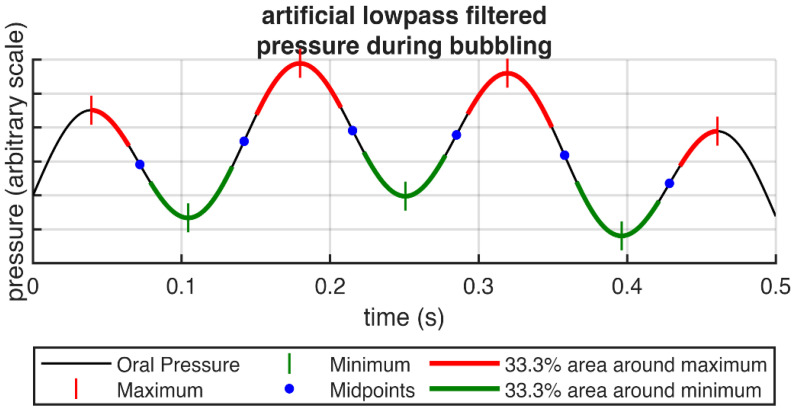
Example illustrating the extraction of lower and upper 33% regions around oral pressure maxima and minima of the pressure signal during bubbling.

The pressure release in the water (in the form of bubbles) leads to a low-frequency pressure fluctuation (i.e., 3–10 Hz). To find a potential influence of high and low pressure on the vocal mechanics, pressure peak and pressure valley areas were extracted from the pressure signal as follows: To retrieve the slow varying pressure signal, the original pressure signal was bandpass filtered using the frequencies 1, 3, 10 and 20 Hz (stopband, passband, passband, stopband frequency). The filter was designed as an IIR Butterworth filter with a stopband attenuation of 60 dB after forward-and-backward filtering (i.e., 30 dB after the first filtering). Forward-and-backward filtering makes sure that no lag is added to the signal and also avoids any frequency phase shifts. The signal was then searched for minima and maxima, using Matlab’s islocalmin() and islocalmax() functions, using a minimum extremum distance corresponding to 10 Hz and a minimum extremum prominence of 10% of the value range of the filtered signal. The prominence of a maximum is the smallest value difference between the maximum and the next neighboring (left and right) minimum (and vice versa for minima). Each of the neighboring minimum-maximum (and maximum-minimum) pairs was then processed separately: the lower 33% area between the two extrema was marked as a pressure valley area, and similarly, the upper 33% area was marked as a pressure peak area (Figure 2). The areas between extrema of the same type were not processed. Finally, OQ_GAW/_, OQ_EGG_ and ClQ_GAW_ were calculated separately for each region type.

**D. Measures**

The HSV video data were segmented using the Glottal Analysis Tools (GAT) Software v.2020 (University Hospital at FAU Erlangen-Nürnberg, Erlangen, Germany) [22], estimating the time-varying lateral deflection of the vocal fold edges along the anterior–posterior glottal dimension [23]. Based on the resulting glottal area waveform, the open quotient (OQ_GAW_) and the closing quotient (ClQ_GAW_, Closing Phase/Period) were calculated for the defined windows using Vocaliscope 3.4 (Jonas Kirsch, LMU University Hospital Munich, Munich, Germany). For OQ_GAW_ computation, a tolerance threshold of 5% was chosen. Consequently, all GAW values > 5% of baseline (i.e., pixel count of the fully open glottis) were considered to indicate an open glottis, while the condition GAW ≤ 5% was used as an indication of a closed glottis.

**E. Statistical Analysis**

The parameters determined for each study participant at two time points were statistically analyzed using WOLFRAM MATHEMATICA (Version 14.0, Champaign, IL, USA (2024)) and MATLAB (Version 24.1.0 (R2025a), Natick, MA, USA: The MathWorks Inc.). The nonparametric Wilcoxon signed-rank test was used to test whether the open and closing quotients of the vowels differed significantly between vowels. The significance level was set at α = 0.05. *p*-values were adjusted for multiple comparisons using the Bonferroni correction (m = 3).

## 3. Results

**Figure 3:**
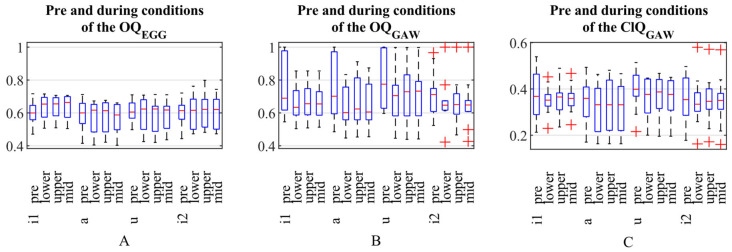
(**A**–**C**): Pre and during conditions for OQ_EGG_ (**A**), OQ_GAW_ (**B**) and ClQ_GAW_ (**C**). The three during WRT conditions (33.3% area around minimum = lower, 33.3% area around maximum = upper, 33.3% area around middle = mid) are shown separately.

Differences in open quotients and closing quotients were observed in the pre conditions among the different vowel qualities. With regard to the GAW, it can be seen that the OQ_GAW_ of the vowel /u/ was significantly higher than the OQ_GAW_ for /a/ (*p* < 0.02), /i1/ (*p* < 0.02) and /i2/ (*p* < 0.006) (Figure 3, Table 2). There was no statistically significant difference between i1 and i2. Similarly, the ClQ_GAW_ for the vowel /u/ was significantly higher than for ClQ_GAW_ /a/ (*p* < 0.01) (Figure 3, Table 3). Electroglottographic measures did not exhibit significant vowel-related differences (corrected *p*-values = 1).

**Figure 4:**
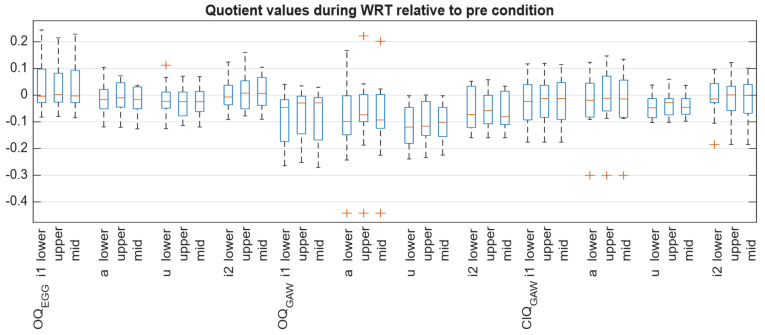
The relationship (presented as quotient values) between the during condition and pre condition. The three during WRT conditions (33.3% area around minimum = lower, 33.3% area around maximum = upper, 33.3% area around middle = mid) are shown separately for OQ_EGG_, OQ_GAW_ and ClQ_GAW_.

**Figure 5:**
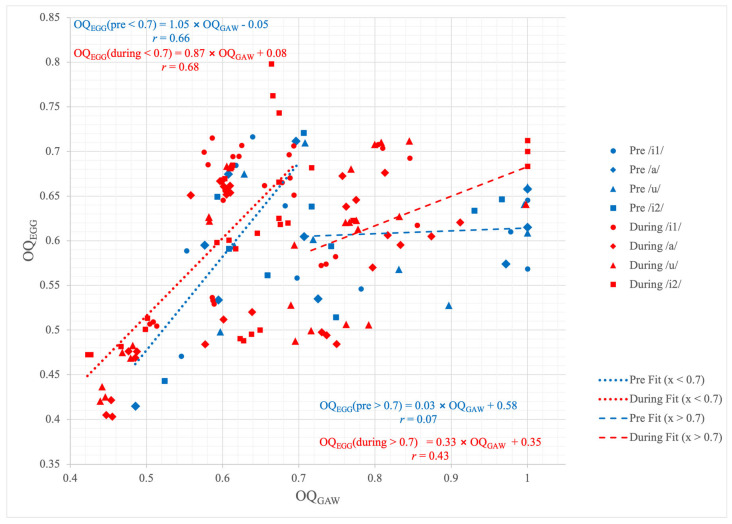
Scatter plot showing the correlation between OQ_GAW_ and OQ_EGG_ for the vowels /i1/, /a/, /u/ and /i2/ in the pre and during conditions. Fitting curves for all OQ_GAW_ values up to 0.7 for the entire pre and during data are plotted.

The OQ_GAW_ showed some minor changes regarding the WRT. Its median value decreased from the pre to the during condition for all vowels, with the largest decrease observed for /u/ (Figure 4). However, these changes failed to show statistical significance. Although the differences among the vowel qualities during WRT were similar to the findings for the pre-condition, i.e., that /u/ had a greater OQ_GAW_ compared to the other vowel conditions, these differences also did not reach a statistically significant level. In contrast to OQ_GAW_, there was a non-significant tendency for OQ_EGG_ to increase the pre-condition to WRT. Concerning OQ_EGG_, there were no substantial changes for the different vowel qualities. As can be seen in Figure 5, there was a good agreement of OQ_GAW_ and OQ_EGG_ for OQ_GAW_ values below 0.7 but a rather strong disagreement for values above. Concerning ClQ_GAW_, the values resembled the changes in OQ_GAW_. Still, also for ClQ_GAW_ the differences were found not statistically different.

**Figure 6:**
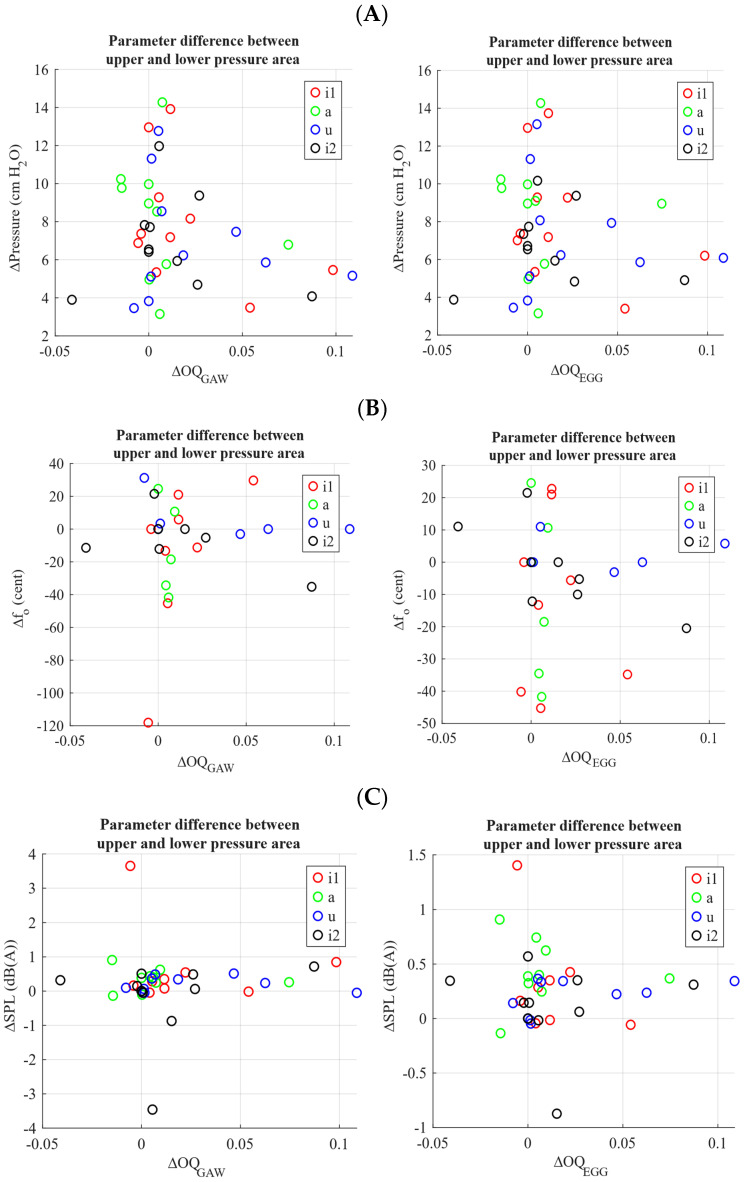
Correlation of OQ_GAW_ and OQ_EGG_ difference (calculation: maximum of 33.3% area around maximum minus minimum of 33.3% area around minimum) with (**A**) pressure, (**B**) fundamental frequency (ƒ_o_) and (**C**) SPL difference.

The pressure difference between maximum and minimum pressure, which should be a value reflecting the size of the bubble, did not show a systematic correlation with the difference in OQ_GAW_ or OQ_EGG_ between maximum and minimum pressure, i.e., that a greater pressure difference would exhibit a greater open quotient difference from the maximum to the minimum pressure (Figure 6A). Neither were there systematic correlations of OQ differences with regard to ƒ_o_ and SPL (Figure 6B,C).

## 4. Discussion

The presented study analyzed changes in vowel quality during a WRT using the DoctorVox^®^ mask. In general, it was found that, although there were differences in vocal fold oscillation patterns for the different vowel qualities during phonation without a mask, no such clear effects were detectable during WRT.

For voice therapy, semi-occluded vocal tract exercises (SOVTE), such as WRT, have been widely used [7]. The great benefit of the DoctorVox^®^ mask compared to tube-based methods such as LaxVox^®^ is that it does not constrain the mouth opening. Consequently, articulation can be achieved in a more physiologically natural manner. From this perspective, one might expect vowel-dependent articulatory differences to influence vocal fold oscillation patterns during WRT.

For phonation without WRT, previous studies have demonstrated that voice source characteristics vary systematically across vowel qualities, reflecting differences in vocal tract configuration and source–filter interaction [23,24,25]. In addition, Fröhlich et al. reported in their validation of the Göttingen Hoarseness Diagram that the vowel /u/ was associated with increased noise components [26]. The increased noise is commonly interpreted as a consequence of stronger turbulent airflow, which in turn may be related to a larger open quotient. In line with these findings, the present dataset showed higher OQ_GAW_ values for /u/ compared to the other vowels, as well as an increase in ClQ_GAW_ relative to /a/. Interestingly, these vowel-dependent differences were no longer clearly observable during WRT. Although small differences in the same manner persisted, none reached statistical significance. Several explanations may account for this observation. One possibility is that the bubbling process itself introduces irregularities that increase the variability of cycle-to-cycle OQ values, thereby masking systematic vowel effects. Alternatively, WRT may lead to a partial equalization of vowel-specific articulation patterns. In this context, the increased pressure during WRT could influence not only vocal fold vibration but also the configuration of the vocal tract, reducing articulatory contrasts between vowel qualities.

A further noteworthy finding was a non-significant tendency for OQ_GAW_ to decrease during WRT compared to the pre-condition. Under conditions of increased pressure, an increase in OQ might be expected, as reported in previous studies [13]. The absence of such an increase, or even a decrease, could therefore indicate the presence of a compensatory strategy during WRT, such as increased adduction. Consistent with this explanation, Laukkanen et al. had shown that the contact quotient in electroglottography (CQ_EGG_) increased during a WRT [27].

Future studies might investigate this hypothesis by tracking the movement of articulation structures, for example, the cuneiform cartilages, as established in articulation research [28]. Within the WRT condition, OQ changes remained small overall; however, during phases of increased pressure within a bubble cycle OQ_GAW_ tended to be higher. At the same time, however, there was no clear tendency that a larger bubble amplitude, as reflected by the greater pressure difference between the maximum and minimum, was associated with a larger change in OQ_GAW_ during the bubble cycle.

Remarkably, OQ_EGG_ exhibited a contrasting behavior compared to OQ_GAW_. It is important to note that GAW and EGG capture different physiological aspects: while GAW reflects the two-dimensional oscillatory behavior of the vocal folds from a superior view, EGG measures impedance changes associated with vocal fold contact. Previous studies have reported good agreement between OQ_GAW_ and OQ_EGG_ for OQ_GAW_ values below 0.7, but substantial divergence for higher values [29]. In the present dataset, there was a comparable finding with good agreement for OQ_GAW_ below 0.7. At the same time, the disagreement between both measures for values above 0.7 was also present; however, it was much more pronounced for phonation without WRT. The underlying reason for the latter observation remains unclear; however, it is conceivable that the altered phonatory conditions during WRT, with the lower OQ_GAW_ during WRT, contributed to this divergence. Several important limitations should be considered when interpreting the results of the present study, which also highlight the need for future investigations to further verify the technical efficacy. First, all measurements were conducted using a water depth of 2 cm; therefore, potential effects of different water depths cannot be excluded. In addition, the use of an endoscope itself may have influenced the phonatory conditions, as it could have introduced partial pressure release within the vocal tract, thereby altering the intended resistance conditions during WRT. Furthermore, the sample size was relatively small (*n* = 10), which limits statistical power. Due to the complexity and extensiveness of the experimental setup, the inclusion of a larger cohort was not feasible. Expanding the sample size in future studies would strengthen the generalizability of the findings. Objective sound pressure level (SPL) control was difficult to achieve under the experimental conditions, particularly during WRT, and therefore could not be implemented with sufficient precision. Finally, the mechanical properties of the DoctorVox^®^ mask should be considered. The mask walls are slightly compliant, which may affect the effective resistance and pressure build-up during phonation. Consequently, the exact magnitude of supraglottal pressure and wall displacement cannot be determined with certainty. Further studies examining different mask designs and material properties are warranted to better understand their impact on the physiological outcomes of the technique.

## 5. Conclusions

In contrast to LaxVox^®^, which limits the mouth opening to a quasi /u/ vowel condition, water resistance therapy using the DoctorVox^®^ mask enables vowel quality differences. However, these differences are attenuated in comparison to differences observed during phonation without WRT with respect to vocal fold oscillation patterns, indicating a partial equalization of oscillatory behavior under conditions of oscillating oral pressure. This suggests that for voice therapy, vowel quality modifications during WRT using the DoctorVox^®^ mask could have little impact on vocal outcomes. The divergences between GAW- and EGG-based OQ values, which change from pre to during condition, show good agreements for OQ values < 0.7 and disagreements for values > 0.7. These findings might contribute to a better understanding of the possible advantages of the DoctorVox^®^ mask and support its use as a therapeutic technique.

## Figures and Tables

**Table 1:** Subjects.

Subject Number	1	2	3	4	5	6	7	8	9	10
**Age (y)**	29	29	46	25	34	25	31	48	26	50
**Sex**	♀	♀	♂	♂	♀	♀	♀	♂	♂	♂

**Table 2:** Wilcoxon signed rank test for OQ_GAW_. Significant values are highlighted in green.

Wilcoxon Signed Rank Test (OQ_GAW_) pre u Tested Against:	*p*-Value	Corrected *p*-Value (Bonferroni Factor 3)
Pre i1	0.008	0.023
Pre a	0.008	0.023
Pre i2	0.002	0.006

**Table 3:** Wilcoxon signed rank test for ClQ_GAW_. Significant values are highlighted in green.

Wilcoxon Signed Rank Test (ClQ_GAW_) pre u Tested Against:	*p*-Value	Corrected *p*-Value (Bonferroni Factor 3)
Pre i1	0.064	0.193
Pre a	0.004	0.0117
Pre i2	0.105	0.316

## Data Availability

Data are available on request.
